# New approaches to tannin analysis of leaves can be used to explain *in vitro* biological activities associated with herbivore defence

**DOI:** 10.1111/nph.16117

**Published:** 2019-09-20

**Authors:** Karen J. Marsh, Ian R. Wallis, Carsten Kulheim, Robert Clark, Dean Nicolle, William J. Foley, Juha‐Pekka Salminen

**Affiliations:** ^1^ Research School of Biology The Australian National University Canberra ACT 2601 Australia; ^2^ Research School of Finance Actuarial Studies and Statistics The Australian National University Canberra ACT 2601 Australia; ^3^ Currency Creek Arboretum PO Box 808 Melrose Park SA 5039 Australia; ^4^ Natural Chemistry Research Group Department of Chemistry University of Turku Turku FI‐20500 Finland

**Keywords:** *Eucalyptus* leaves, herbivory, hydrolysable tannins, nitrogen digestibility, oxidative activity, polyphenols, proanthocyanidins, protein precipitation capacity

## Abstract

- Although tannins have been an important focus of studies of plant–animal interactions, traditional tannin analyses cannot differentiate between the diversity of structures present in plants. This has limited our understanding of how different mixtures of these widespread secondary metabolites contribute to variation in biological activity.
- We used UPLC‐MS/MS to determine the concentration and broad composition of tannins and polyphenols in 628 eucalypt (*Eucalyptus*,* Corymbia* and *Angophora*) samples, and related these to three *in vitro* functional measures believed to influence herbivore defence: protein precipitation capacity, oxidative activity at high pH and capacity to reduce *in vitro* nitrogen (N) digestibility.
- Protein precipitation capacity was most strongly correlated with concentrations of procyanidin subunits in proanthocyanidins (PAs), and late‐eluting ellagitannins. Capacity to reduce *in vitro* N digestibility was affected most by the subunit composition and mean degree of polymerisation (mDP) of PAs. Finally, concentrations of ellagitannins and prodelphinidin subunits of PAs were the strongest determinants of oxidative activity.
- The results illustrate why measures of total tannins rarely correlate with animal feeding responses. However, they also confirm that the analytical techniques utilised here could allow researchers to understand how variation in tannins influence the ecology of individuals and populations of herbivores, and, ultimately, other ecosystem processes.

## Introduction

Biologists often assume that a primary role of tannins is to defend plants against herbivory. There are, however, thousands of tannin compounds displaying a diverse array of structures that often occur in complex mixtures of tens to hundreds of compounds. These structures not only influence the response to standard colorimetric assays (Schofield *et al*., 2001), but they also affect the biological activity of tannins, including their ability to bind proteins (Porter & Woodruffe, 1984; Jones & Palmer, 2000; Karonen *et al*., 2015), their pro‐ or antioxidant capacities (Barbehenn *et al*., 2006; Moilanen & Salminen, 2008; Moilanen *et al*., 2016), and, ultimately, their effects on herbivores (Ayres *et al*., 1997; Makkar, 2003; Mueller‐Harvey, 2006; Roslin & Salminen, 2008). Consequently, the specific tannins present in a mixture, rather than just the total tannin concentration, are important in understanding biological consequences (Barbehenn *et al*., 2008; Moilanen & Salminen, 2008). However, it is difficult to characterise complex tannin mixtures, and therefore to attribute specific biological consequences to tannins. Some ecologists have circumvented this problem by relating measures of herbivory to functional attributes of tannins, such as their capacity to precipitate protein (Robbins *et al*., 1987; e.g. McArt *et al*., 2009), reduce nitrogen (N) digestibility (e.g. DeGabriel *et al*., 2009; McArt *et al*., 2009) or oxidise at high pH (e.g. Appel, 1993; Steinbauer *et al*., 2016; Marsh *et al*., 2017b).

Protein binding is the primary mechanism by which tannins are traditionally thought to affect mammalian herbivores. Thus, ecologists measure the capacity of tannins to precipitate a standard amount of protein and use this as a functional measure of tannin concentrations in forage. For example, Robbins *et al*. (1987) were able to predict the *in vivo* digestible protein content of plants for mule deer (*Odocoileus hemionus*) and white‐tailed deer (*O. virginianus*) from the *in vitro* protein precipitation capacity and total protein concentration of plant extracts. In turn, McArt *et al*. (2009) demonstrated that reduced protein availability due to protein precipitation by tannins could explain differences in productivity between moose (*Alces alces*) living in different regions.

DeGabriel *et al*. (2008) developed an alternative method to assess the capacity of tannins to constrain protein digestion in mammalian herbivores. Their method measures the *in vitro* digestibility of plant N in the presence and absence of polyethylene glycol (PEG), a polymer that preferentially binds to tannins and releases protein (Silanikove *et al*., 1996; Schofield *et al*., 2001). Using this method, DeGabriel *et al*. (2009) showed that the *in vitro* digestible N concentration of eucalypt foliage influenced the reproductive success of female common brushtail possums (*Trichosurus vulpecula*). As a consequence, *in vitro* digestible N concentrations have been used as indicators of the nutritional quality of habitat for marsupial folivores (DeGabriel *et al*., 2009; Youngentob *et al*., 2011; Windley *et al*., 2016).

In contrast to mammals, there is little evidence that tannins reduce the digestibility of protein in insect herbivores (Barbehenn & Constabel, 2011). The alkaline pH in the midgut of insects can prevent tannins from forming complexes with protein (Martin *et al*., 1985; Appel, 1993; Barbehenn & Constabel, 2011). This is not to say that tannins are harmless to insects. Instead, the alkaline conditions may promote the oxidation of tannins and other phenolic compounds, leading to the formation of oxygen radicals and, consequently, cell damage (Appel, 1993). For example, the larvae of some tropical lepidopteran species were found to contain the oxidation products of polyphenols in their frass (Vihakas *et al*., 2015). Likewise, *Lymantria dispar, Orgyia Leucostigma* and *Malacosoma distria* caterpillars that fed on maple leaves with high concentrations of ellagitannins had high levels of semiquinone radicals in their midguts together with increased protein carbonyl contents that suggested increased oxidation of the proteins in the gut (Barbehenn *et al*., 2005, 2013).

The above examples demonstrate that measuring the activity of polyphenol mixtures has provided a better indication of their probable effects in different biological systems compared with estimates of ‘total tannins’ or ‘total phenolics’. Fortunately, new analytical techniques are now available that allow the broad characterisation of complex polyphenol mixtures in plants. For example, as part of a related study on the phylogeny of tannins in eucalypts, we used ultraperformance liquid chromatography tandem mass spectrometry (UPLC‐MS/MS, i.e. the Engström method; Engström *et al*., 2014, 2015; Salminen, 2018b) to measure the concentrations of a range of phenolic subgroups, including four subgroups of tannins, in leaves from 628 eucalypts representing 515 species (Marsh *et al*., 2017a). This provides an ideal opportunity to examine the relationship between plant polyphenol composition and traditional *in vitro* measures of biological activity. Knowing what groups of tannins are active also improves our ability to identify the genetic architecture of tannin production. For example, Skovmand *et al*. (2018) recently argued that genes responsible for variations in tannins in plants may be ‘keystone genes’ that are critical to ecosystem function.

Eucalypts are the dominant forest and woodland trees in Australia, with *c*. 900 species belonging to the genera *Eucalyptus*,* Angophora* and *Corymbia* (Bayly *et al*., 2013). Eucalypt foliage makes up a large proportion of the diet of four species of marsupials – the koala (*Phascolarctos cinereus*), greater glider (*Petauroides volans*), common ringtail possum (*Pseudocheirus peregrinus*) and common brushtail possum (*Trichosurus vulpecula*) (Moore *et al*., 2004) – and a wide variety of insect herbivores (Fox & Morrow, 1981; Paine *et al*., 2011). Importantly, eucalypts contain variable concentrations and types of tannins and other polyphenols, suggesting that there may also be significant variation in biological activity associated with herbivore defence (Marsh *et al*., 2017a).

We had several expectations about how specific tannin subgroups would influence traditional measures of *in vitro* biological activity (protein precipitation capacity, oxidative activity and capacity to reduce digestible N) of eucalypt samples (Table 1). However, we also understood that our hypotheses are complicated by the fact that individual tannins within the same subgroup can differ over six‐fold in both their protein precipitation (Karonen *et al*., 2015) and their oxidative activities (Moilanen & Salminen, 2008). Thus, differences in the types and concentrations of individual tannins between eucalypt species could influence the degree of the response to the standard assays. Nevertheless, we expected to reveal the major patterns between the tannin groups and their bioactivities, and hoped to see some of the more detailed patterns within the tannin subgroups.

**Table 1:** The polyphenol constituents measured in this study, and their expected effects on biological activity.

Constituent	Polyphenol class	Expected effect on biological activity		
		PPC	OA	CND
Small procyanidin	PA	+		+
Medium procyanidin	PA	+		+
Large procyanidin	PA	++		++
Small prodelphinidin	PA	+	+	+
Medium prodelphinidin	PA	++	+	++
Large prodelphinidin	PA	+++	+	++
% prodelphinidin	PA	+	+	+
mDP	PA	++		+
Early‐eluting HHDP derivatives	HT		+++	
Late‐eluting HHDP derivatives	HT	+	++	
Early‐eluting galloyl derivatives	HT		++	
Late‐eluting galloyl derivatives	HT	++	+	
Kaempferol derivatives	Flavonol			
Quercetin derivatives	Flavonol			
Myricetin derivatives	Flavonol		+	
Quinic acid derivatives	Flavonoid		+	

A ‘+’ indicates an expected positive relationship, with ‘++’ and ‘+++’ indicating the constituents hypothesised to have the strongest effects on biological activity.

PA, proanthocyanidin; HT, hydrolysable tannin; PPC, protein precipitation capacity (mg g^−1^ DM pentagalloyl glucose equivalents); OA, oxidative activity (mg g^−1^ DM gallic acid equivalents); CND, capacity to reduce N digestibility (percentage units); mDP, mean degree of polymerisation of proanthocyanidins; HHDP, hexahydroxydiphenoyl.

We anticipated that protein precipitation capacity would be positively correlated with the concentrations of proanthocyanidins (PAs; also known as condensed tannins), their mean degree of polymerisation (mDP) and the proportion of prodelphinidin subunits in PAs (Jones *et al*., 1976; Porter & Woodruffe, 1984; McManus *et al*., 1985; Aerts *et al*., 1999). At a finer scale, we used the Engström method (Engström *et al*., 2014) to measure PA concentrations at three cone voltages (see Salminen, 2018b), which provides additional information about the size distribution of PAs. We expected that large prodelphinidin‐rich PAs in particular would contribute positively to the protein precipitation capacity of samples, assuming that these types of PAs were in sufficient concentrations in the extracts. Because most of the galloyl groups in our samples originate from monomeric ellagitannins or simple galloyl glucoses, rather than gallotannins (Marsh *et al*., 2017a), and because gallotannins have a better protein precipitation capacity compared with monomeric ellagitannins or simple galloyl glucoses (Haslam, 1988; Kawamoto *et al*., 1996; Kilkowski & Gross, 1999; Salminen & Karonen, 2011), we did not expect a strong correlation between protein precipitation capacity and galloyl‐derivative concentrations as such, unless the galloyl derivatives were the main tannins of the species.

We hypothesised that the properties of tannins that affect protein precipitation capacity would also influence their capacity to reduce *in vitro* N digestibility. The gut contains both exogenous protein from the diet and endogenous proteins, such as enzymes, sloughed mucosal cells and microbial protein. Tannins that precipitate any of these proteins will reduce the apparent digestibility of N. We therefore expected that the concentration, size and composition of PA molecules would strongly influence the capacity to reduce *in vitro* digestible N, and that there would be a positive correlation between protein precipitation capacity and capacity to reduce *in vitro* N digestibility in eucalypts.

In contrast to the major role that PAs may play in influencing protein precipitation capacity and capacity to reduce N digestibility, we predicted that the concentration of hexahydroxydiphenoyl (HHDP) derivatives (i.e. ellagitannins), particularly those that elute earlier during UPLC separation, would drive the oxidative activity of eucalypt samples, if they are present at sufficient concentrations compared to other oxidatively active compounds in the samples. This is because ellagitannins appear to be the class of tannins that are most oxidatively active at high pH (Barbehenn *et al*., 2006; Moilanen & Salminen, 2008), and those with shorter UPLC retention times tend to oxidise more readily than those with longer retention times (Salminen *et al*., 2011; Moilanen *et al*., 2013). Other polyphenol constituents, including prodelphinidin subunits of PAs and myricetin derivatives, with pyrogallol‐type substitution of the flavonoid B‐ring, may also contribute to oxidative activity to a lesser extent (Vihakas *et al*., 2014).

Our final prediction was that there would be a negative correlation between oxidative activity and protein precipitation capacity. This prediction was made for two reasons. First, hydrolysable tannins (HTs) with high protein precipitation capacity tend to have lower oxidative activity (Moilanen *et al*., 2013). And second, there is an inverse relationship between the concentrations of PAs and HTs in eucalypt leaves, probably because they compete for biosynthetic pathways (Marsh *et al*., 2017a). Given the expected reciprocal effects of PAs and HTs on oxidative activity and protein precipitation capacity, protein precipitation capacity may be higher in those samples dominated by PAs, while samples dominated by HTs may be more oxidatively active.

## Materials and Methods

The collection of 628 leaf samples (515 eucalypt species from the allied genera *Eucalyptus* L'Her., *Corymbia* Hill & Johnson and *Angophora* Cav. – duplicates of species were predominantly different subspecies) from Currency Creek Arboretum, South Australia, and the measurement of the phenolic composition of these samples by UPLC‐MS/MS is described in detail in Marsh *et al*. (2017a). Hydrolysable tannins (HHDP and galloyl derivatives), however, were re‐integrated to determine concentrations of early‐eluting and late‐eluting derivatives for each group. We used the dimeric ellagitannin, oenothein B, as a marker to distinguish the difference between these two groups. The early‐eluting HHDP and galloyl derivatives eluted before oenothein B (0.5–2.9 min), and the late‐eluting ones were those from oenothein B onwards (2.9–6 min). Likewise, we re‐analysed the previously collected data to determine the concentration of procyanidins and prodelphinidins in each of the three size classes explained in Salminen (2018b). The smaller procyanidin and prodelphinidin oligomers were detected by cone voltages of 75 and 55 V, the medium procyanidin and prodelphinidin oligomers and polymers by cone voltages of 85 and 80 V, and the large procyanidin and prodelphinidin polymers by cone voltages of 140 and 130 V, respectively. A summary of the phenolic constituents that were measured is given in Table 1, along with their expected effects on biological activity.

### Protein precipitation capacity

The protein precipitation capacity of eucalypt extracts was quantified by the radial diffusion assay (Hagerman, 1987) using BSA as the protein and pentagalloylglucose as the quantification standard. Briefly, the original, nondiluted eucalypt extract (Marsh *et al*., 2017a) was concentrated two‐fold via freeze‐drying and redissolving into Milli‐Q water. A 24 μl aliquot of this concentrated extract was applied to three wells punched onto a Petri dish filled with BSA‐agar gel. The Petri dishes were covered with parafilm and incubated at 30°C for 72 h to form reproducible rings with tannins and BSA. The ring area was documented with a camera on a tripod and measured by the imageJ software.

### Oxidative activity

The portion of total phenolics that was easily auto‐oxidised at pH 10 was measured as both mg g^−1^ dry weight and as a percentage of total phenolics using the method of Salminen & Karonen (2011), calibrated with gallic acid. In short, the total phenolic content of 15× dilutions (Milli‐Q water) of the original eucalypt extracts (Marsh *et al*., 2017a) and the pH 10‐oxidised extracts were measured with a well‐plate reader at 730 nm. The difference in the total phenolic concentrations between these measurements revealed the level of easily oxidised phenolics in the samples (i.e. the oxidative activity in mg g^−1^ or in % of total phenolics).

### Capacity to reduce *in vitro* N digestibility

A subset of leaves from each tree were freeze dried and then ground in a Foss Cyclotec 1093 mill (Foss, Höganäs, Sweden) until they passed through a 1 mm sieve. The capacity to reduce *in vitro* N digestibility was determined using a modified version of the method of DeGabriel *et al*. (2008). The method involves sequential digestion of samples of ground foliage in acid pepsin and cellulase in the presence and absence of polyethylene glycol 4000 (PEG), which binds to both HTs and PAs (Silanikove *et al*., 1996; Schofield *et al*., 2001).

For each sample, 0.8050 ± 0.0050 g leaf powder was weighed into each of four Ankom F57 fibre filter bags (Ankom Technology, Macedon, NY, USA). Two bags per sample were placed into beakers (100 bags per beaker) containing 25 ml Tris‐base buffer solution (pH 7.1) per bag with 33.33 g l^−1^ PEG. The remaining bags were placed into the buffer solution without PEG. Samples were incubated at 37°C for 24 h, after which they were washed thoroughly with water and dried to constant mass at 40°C. Bags were then placed into 25 ml per bag of 0.1 M HCl containing 2 g l^−1^ pepsin for 48 h at 37°C. Samples were removed from the pepsin solution and washed briefly, before a final incubation at 37°C for 24 h in 25 ml per bag of 100 mmol acetic acid buffer (pH 4.75) containing 6.25 g l^−1^ cellulase. Samples were washed thoroughly, dried at 40°C to constant mass and weighed.

After the digestion process, the N concentration was determined in 120 ± 20 mg of residue from each bag, as well as in the original ground leaf samples, using a Leco Truspec C/N analyser (Leco Corporation, Sydney, NSW, Australia). These values were used to calculate the *in vitro* digestibility of N in the presence and absence of PEG. Any samples with a coefficient of variation > 5% between duplicate analyses were repeated. The capacity to reduce *in vitro* N digestibility was calculated as the difference in N digestibility between samples incubated with and without PEG. The digestibility with PEG was used rather than total N, because the total N of a plant will never be completely digested, due to some of the N being bound in complex cell‐wall polymers.

### Statistical methods

We compared the mean biological activity (protein precipitation capacity, oxidative activity and capacity to reduce *in vitro* N digestibility) of eucalypt phylogenetic clades (see Marsh *et al*., 2017a for the allocation of species to clades) using the gls function in package nlme for R (Pinheiro *et al*., 2017) and Pagel's λ covariance structure (R package ape; Paradis *et al*., 2004) to account for the phylogenetic nonindependence of samples. For all analyses, the dataset and analyses were at the individual tree level, with multiple subspecies and trees observed for some species, while other species were observed only once. Pagel's λ model is equivalent to including a within‐species error term in addition to phylogenetic between‐species term, and so is suitable for data where there are multiple observations per species. We used Tukey contrasts to determine which clades differed significantly (*P *<* *0.05) from one another. Residuals were checked for normality and variance homogeneity in all models.

We estimated correlations between protein precipitation capacity and oxidative activity, protein precipitation capacity and capacity to reduce *in vitro* N digestibility, and oxidative activity and capacity to reduce *in vitro* N digestibility using the corphylo function in the ape package in R to take the phylogenetic relatedness of species into account. Standard errors and *t*‐statistics for the estimated correlations were calculated using a parametric bootstrap with 30 replicates under the null hypothesis of independence, using simple phylogenetic regression models fitted using the gls function in the ape package in R.

We also used the gls function to fit four regression models for each of the square root of protein precipitation capacity, oxidative activity and the square root of the capacity to reduce N digestibility, while taking the phylogeny into account. The square root transformation was applied to two of these three dependent variables in order to better satisfy the assumption of normally distributed errors with equal variances. The first model for each dependent variable contained the intercept only. The second model contained the total polyphenol concentration (the sum of all measured constituents). The third model contained two terms: total tannins (the sum of all constituents in the PA and HT classes; Table 1) and total flavonols (the sum of all constituents in the flavonol class; Table 1). The fourth model contained all individual constituents listed in Table 1. We tested the significance of the fourth model using likelihood ratio tests relative to the second and third models, which are both submodels of it.

To explore which constituents were most important in predicting biological activity, we performed a backward selection of all of the covariates in the fourth model outlined in the previous paragraph for each of the three transformed activities based on the significance of omitting variables as calculated using a chi‐squared likelihood ratio test with a cutoff of 0.05 for significance. So that models made biological sense, either the total concentration of prodelphinidin (sum of the concentrations of prodelphinidin from small, medium and large PAs; Table 1) was allowed in the model or one or more of the separate small, medium or large concentrations of prodelphinidin. Combinations of total prodelphinidin with prodelphinidin subgroups were not allowed. The total procyanidin (or small, medium and large), total HHDP derivatives (or early and late) and total gallic acid derivatives (or early and late) variables were treated similarly. Residuals were checked for normality and homogeneity of variances, and six outliers with high leverage were removed. These included two samples with oxidative activity and three samples with protein precipitation capacity almost double that of the next highest samples, and a sample which turned out to be an outlier in regression models of *in vitro* N digestibility.

## Results

### *In vitro* polyphenol‐derived biological activity

We found wide variation in the protein precipitation capacity (0–229 mg g^−1^ dry matter (DM) pentagalloyl glucose equivalents), oxidative activity (2–94 mg g^−1^ DM gallic acid equivalents, or 3–79% of total phenolics) and capacity to reduce *in vitro* N digestibility (0–89 percentage units) between eucalypt species (Table 2). Mean biological activity did not differ between phylogenetic clades when the relatedness of species was taken into account (Table 2).

**Table 2:** The mean (range) biological activity of species belonging to different eucalypt clades.

Phylogenetic clade	*n*	PPC^a^	OA^b^	CND^c^
*Angophora*	5	25 (9–59)	21 (6–40)	19 (8–44)
*Corymbia* I	19	18 (0–40)	16 (5–30)	29 (11–45)
*Corymbia* II	13	28 (5–53)	19 (6–34)	24 (13–38)
*Eudesmia*	10	42 (6–92)	29 (10–50)	40 (1–89)
*Monocalyptus*	79	36 (0–122)	19 (4–56)	25 (0–70)
*Symphyomyrtus* I	32	25 (0–89)	12 (2–51)	10 (0–22)
*Symphyomyrtus* II	97	26 (0–98)	19 (3–48)	11 (0–68)
*Symphyomyrtus* III	125	43 (0–229)	22 (2–86)	9 (0–38)
*Symphyomyrtus* IV	76	30 (0–113)	22 (2–94)	28 (0–67)
*Symphyomyrtus* V	166	40 (0–201)	20 (3–50)	10 (0–58)
Pagel's λ		0.46	0.77	0.82
*F*‐statistic		0.99	1.65	1.60
*P*‐value		0.453	0.091	0.103

a Protein precipitation capacity (mg g^−1^ DM pentagalloyl glucose equivalents).

b Oxidative activity (mg g^−1^ DM gallic acid equivalents).

c Capacity to reduce N digestibility (percentage units).

The capacity to reduce *in vitro* N digestibility was not correlated with either the oxidative activity (*t *=* *−0.95, *P *=* *0.341) or protein precipitation capacity of eucalypt leaves (*t *=* *0.36, *P *=* *0.719). However, there was a positive correlation between the protein precipitation capacity and oxidative activity of samples (*r *=* *0.54*, t *=* *3.90, *P *<* *0.001).

### Polyphenol composition and protein precipitation capacity

The model containing all of the polyphenol constituents in Table 1 explained significantly more of the variation in the square root of protein precipitation capacity compared with models that contained only the total polyphenol concentration or a combination of the total tannin and total flavonol concentrations (*P *<* *0.001 for both model comparisons).

**Figure 1:**
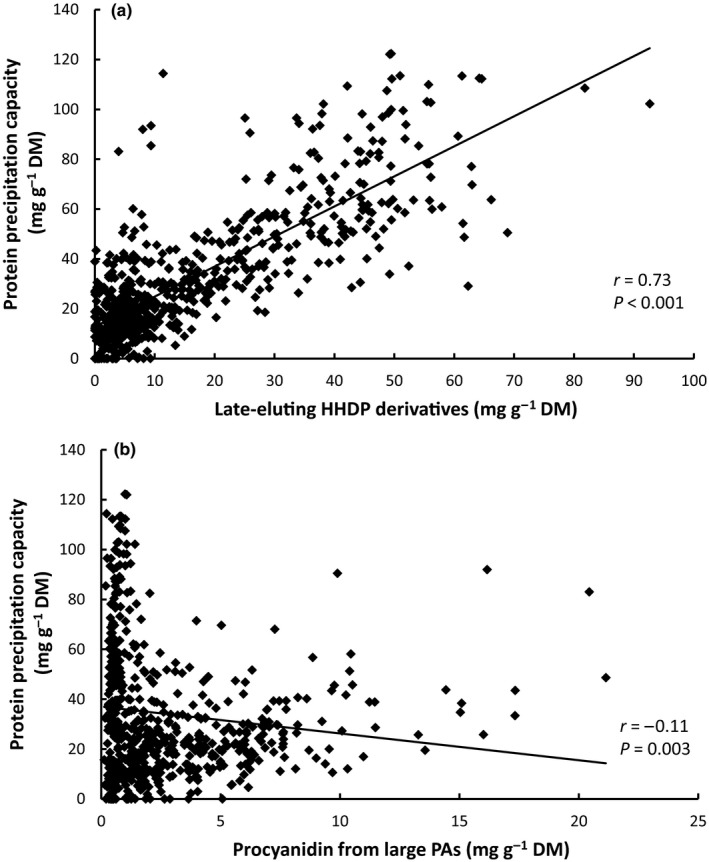
The relationship between the protein precipitation capacity of eucalypt leaves and the two polyphenol constituents that had the strongest correlation with this measurement (*n* = 628): (a) late‐eluting hexahydroxydiphenoyl (HHDP) derivatives, and (b) procyanidin subunits from polymeric proanthocyanidins (PAs). Note that these relationships are indicative only, because they do not take into account other covariates or phylogenetic correlations from the statistical model.

There were strong positive relationships between protein precipitation capacity and the concentrations of late‐eluting HHDP derivatives (Fig. 1a), procyanidin subunits from polymeric PAs (Fig. 1b), galloyl derivatives and prodelphinidin subunits from medium‐sized PAs (Table 3). Although the relationships were not as strong, the concentration of early‐eluting HHDP derivatives and the mDP of PAs were negatively correlated with protein precipitation capacity (Table 3). Pagel's lambda for the model was 0.31 (likelihood ratio χ^2^ = 17.3 on 1 degree of freedom, *P* < 0.001).

**Table 3:** Final statistical model showing the phenolic constituents that had a significant effect on the square root of protein precipitation capacity.

Model term	Parameter estimate	SE	*t* statistic	*P*‐value	Standardised coefficient
(Intercept)	2.393	0.439	5.45	< 0.001	
Late HHDP	0.120	0.006	18.94	< 0.001	0.882
Large procyanidin	0.219	0.018	11.89	< 0.001	0.298
Total galloyl	0.056	0.006	8.75	< 0.001	0.235
Medium prodelphinidin	0.178	0.022	8.17	< 0.001	0.209
Early HHDP	−0.027	0.010	−2.73	0.007	−0.112
mDP of PAs	−0.047	0.018	−2.59	0.010	−0.070

Degrees of freedom for all *t*‐statistics is 563 (*n* = 625).

HHDP, hexahydroxydiphenoyl derivatives; mDP of PAs, mean degree of polymerisation of proanthocyanidins.

### Polyphenol composition and oxidative activity

The full model containing all measured polyphenol constituents explained significantly more variation in oxidative activity compared with either the total polyphenol concentration alone, or a combination of the total tannin and total flavonol concentrations, with *P *<* *0.001 for both model comparisons.

**Figure 2:**
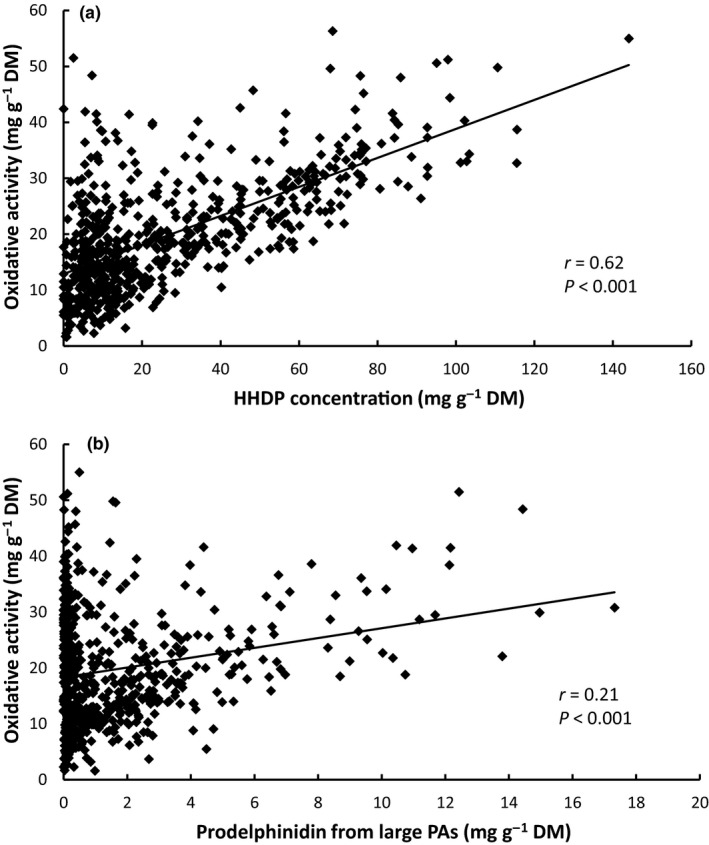
The relationship between the oxidative activity of eucalypt leaves and the two polyphenol constituents that had the strongest correlation with this measurement (*n* = 628): (a) hexahydroxydiphenoyl (HHDP) derivatives, and (b) prodelphinidin subunits from large proanthocyanidins (PAs). Note that these relationships are indicative only, because they do not take into account other covariates or phylogenetic correlations from the statistical model.

The total concentration of HHDP derivatives had a very strong effect on the oxidative activity of samples (Fig. 2a), while there were also strong positive relationships between the oxidative activity of samples and the concentrations of prodelphinidin subunits from large PAs (Fig. 2b), and early‐eluting galloyl derivatives (Table 4). The mDP of PAs and the concentrations of quercetin and quinic acid derivatives were also positively correlated with oxidative activity, while the concentration of prodelphinidin subunits from medium‐sized PAs was negatively correlated (Table 4). Pagel's lambda for the model was 0.58 (likelihood ratio χ^2^ = 21.9 on 1 degree of freedom, *P* < 0.001).

**Table 4:** Final statistical model showing the phenolic constituents that had a significant effect on oxidative activity.

Model term	Parameter estimate	SE	*t* statistic	*P*‐value	Standardised coefficient
(Intercept)	6.656	3.344	1.99	0.047	
Total HHDP	0.303	0.012	24.78	< 0.001	0.763
Large prodelphinidin	2.801	0.420	6.67	< 0.001	0.737
Early galloyl	0.329	0.086	3.84	< 0.001	0.113
mDP of PAs	0.235	0.092	2.56	0.011	0.079
Medium prodelphinidin	−1.015	0.411	−2.47	0.014	−0.271
Quercetin	0.259	0.111	2.33	0.020	0.063
Quinic acid	0.273	0.125	2.19	0.029	0.067

Degrees of freedom for all *t*‐statistics is 563 (*n* = 626).

HHDP, hexahydroxydiphenoyl derivatives; mDP of PAs, mean degree of polymerisation of proanthocyanidins.

### Polyphenol composition and capacity to reduce *in vitro* N digestibility

The full model containing all measured polyphenol constituents explained significantly more of the variation of the square root of the capacity to reduce *in vitro* N digestibility compared with models containing only the total polyphenol concentration, or a combination of the total tannin and total flavonol concentrations (*P *<* *0.001 for both model comparisons).

**Figure 3:**
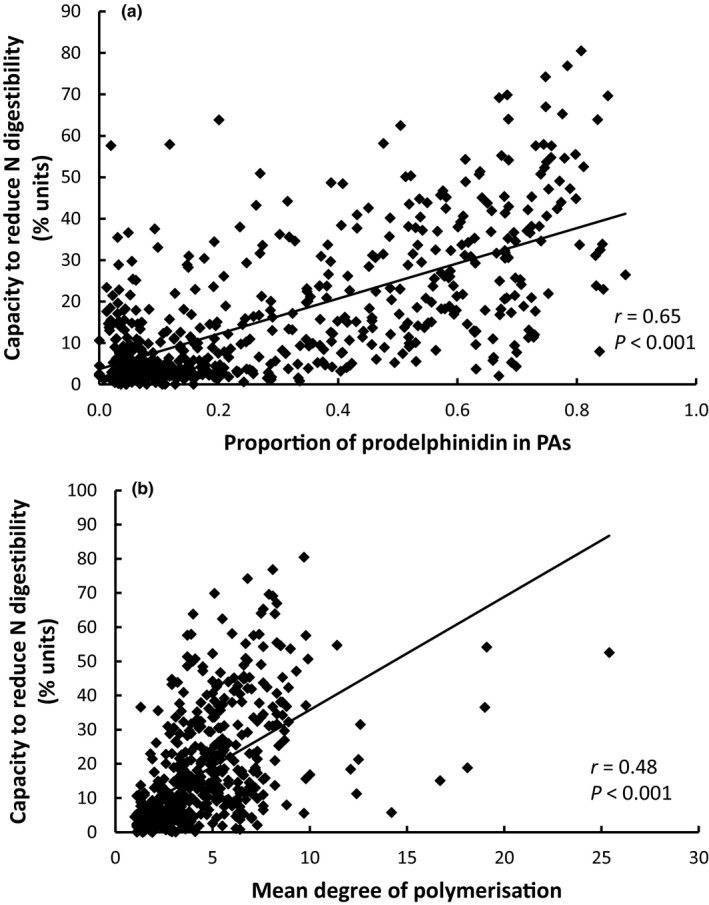
The relationship between the capacity to reduce the *in vitro* nitrogen (N) digestibility of eucalypt leaves and (a) the proportion of proanthocyanidins (PAs) comprising prodelphinidin, and (b) the mean degree of polymerisation (mDP) of proanthocyanidins (*n* = 628). Note that these relationships are indicative only, because they do not take into account other covariates or phylogenetic correlations from the statistical model.

There were strong positive relationships between the capacity to reduce *in vitro* N digestibility and the proportion of prodelphinidin in PAs (Fig. 3a), and the mDP of PAs (Fig. 3b; Table 5). The concentration of PD in small PAs had a strong negative effect on the capacity to reduce *in vitro* N digestibility (Table 5). There was a weaker negative relationship between the capacity to reduce *in vitro* N digestibility and the concentration of quercetin derivatives (Table 5). Pagel's lambda for the model was 0.66 (likelihood ratio χ^2^ = 72.0 on 1 degree of freedom, *P* < 0.001).

**Table 5:** Final statistical model showing the phenolic constituents that significantly influenced the square root of the capacity to reduce *in vitro* N digestibility.

Model term	Parameter estimate	SE	*t* statistic	*P‐*value	Standardised coefficient
(Intercept)	5.883	0.831	7.08	< 0.001	
% prodelphinidin	3.244	0.308	10.54	< 0.001	0.420
mDP of PAs	0.294	0.028	10.37	< 0.001	0.503
Small prodelphinidin	−0.217	0.023	−9.35	< 0.001	−0.376
Quercetin	−0.061	0.025	−2.48	0.014	−0.075

Degrees of freedom for all *t*‐statistics is 566 (*n* = 627).

mDP of PAs = mean degree of polymerisation of proanthocyanidins.

## Discussion

The current study demonstrates that quantifying tannin subgroups in complex polyphenol mixtures provides valuable information about the biological activity of plant samples. The subgroup composition of tannins in eucalypt extracts explained significantly more of the variation in oxidative activity, protein precipitation capacity and capacity to reduce *in vitro* N digestibility compared with the sum of the concentrations of the measured constituents. This is not necessarily surprising, because eucalypt species with the same total polyphenol concentration can have vastly different polyphenol profiles (Marsh *et al*., 2017a). Nevertheless, researchers frequently (and often unsuccessfully) attempt to relate total tannin concentrations to, for example, various measures of herbivory (Ayres *et al*., 1997; Wang *et al*., 2014; Masette *et al*., 2015; Volf *et al*., 2015; Felton *et al*., 2018).

Different tannin subgroups influenced different types of biological activity, although not all of these correlations matched our expectations. For example, concentrations of late‐eluting HHDP derivatives (ellagitannins) were more strongly correlated with protein precipitation capacity compared with concentrations of PA subunits. It is likely, however, that high concentrations of ellagitannins relative to PAs drove this association. Likewise, the size and composition of PAs, rather than their concentration, correlated most strongly with the capacity to reduce *in vitro* N digestibility. As expected, however, the concentration of ellagitannins had the greatest effect on the oxidative activity of samples. This study significantly advances our understanding of structure–function relationships in natural plant tannin mixtures, and demonstrates that modern analytical techniques should be incorporated into studies examining relationships between tannins and herbivory. Below, we discuss the relationships between phenolic composition and biological activity in greater detail, and the implications for herbivores consuming foliage.

Protein precipitation by tannins has been advocated as a mechanism by which plants can defend themselves against a variety of mammalian herbivores, including against those that feed on eucalypt foliage (Marsh *et al*., 2003; DeGabriel *et al*., 2009). At equal concentrations, in a broad sense, PAs have a greater capacity than gallotannins or ellagitannins to precipitate protein, although accurate compound‐specific studies with PAs are scarce (Haslam, 1988; Kilkowski & Gross, 1999; Salminen & Karonen, 2011). The tannin profiles of many of the eucalypts that we analysed were dominated by ellagitannins, with some having concentrations > 100 mg g^−1^ (Fig. 2a; Marsh *et al*., 2017a). In this situation, higher concentrations may compensate for lower bioactivity, and can make a significant contribution to protein precipitation capacity (Johnson *et al*., 2014). This probably explains why the protein precipitation capacity of eucalypt extracts was most strongly correlated with the concentration of late‐eluting HHDP derivatives, and then secondarily with the concentrations of procyanidin subunits in large PAs and prodelphinidin subunits in medium‐sized PAs.

Our finding that the concentration of late‐eluting HHDP derivatives had a greater impact on protein precipitation capacity compared with early‐eluting derivatives supports previous work that individual ellagitannins can differ greatly in their capacity to precipitate protein (Salminen *et al*., 2011; Moilanen *et al*., 2013). Purified ellagitannins with greater protein precipitation capacity tend to elute later during reversed‐phase LC analyses due to their greater structural flexibility, lower water solubility and higher molecular mass (Salminen *et al*., 2011; Moilanen *et al*., 2013; Karonen *et al*., 2015; Engström *et al*., 2019). The following four structural features primarily increase the protein precipitation capacity and retention time of ellagitannins: the number of galloyl, HHDP and other functional groups attached to the central glucose core; the presence of two galloyls instead of one HHDP that is formed by C–C coupling of the two galloyls; the presence of a central glucopyranose unit instead of acyclic glucose; and the oligomerisation degree of the ellagitannin (Engström *et al*., 2019). Thus, separately integrating early‐ and late‐eluting ellagitannins in complex mixtures of unidentified ellagitannins could provide useful information about the proportional biological activity of these compounds. In addition, their elution profiles could give a hint of their structures that could be then verified by UV and MS spectra (Moilanen *et al*., 2013).

Quantifying PAs within different size classes (i.e. the Engström method; Engström *et al*., 2014; Salminen, 2018b) could also improve our understanding of how different mixtures of PAs affect biological activity. The statistical models for all three biological activities identified correlations between biological activity and specific size classes of PA subunits, rather than total concentrations. This suggests that we need a better understanding of the biological activities of individual PAs, and again illustrates why understanding the relationship between biological activity and tannins is so difficult in plant samples; variation in tannin composition, even within subgroups of tannins, influences biological activity.

Some of the eucalypt samples in our study possessed very high oxidative activity (up to 94 mg g^−1^ DM gallic acid equivalents). This is at the higher end of values that have been reported in other plant species (Vihakas *et al*., 2014, 2015). In theory, the oxidative capacity of polyphenols could affect plant resistance to insect herbivory (Appel, 1993), but this has yet to be demonstrated conclusively. Oxidative polyphenols may also be more effective against some insect species than against others. For example, in two species of lepidopteran larvae, *Agriopis aurantiaria* was much more efficient than *Epirrita autumnata* in metabolising pentagalloylglucose, a model hydrolysable tannin (Salminen, 2018a). Interestingly, even though *A. aurantiaria* appears to have a higher gut pH than *E. autumnata* (Kim *et al*., 2018), pentagalloylglucose was not more harmful to *A. aurantiaria*. While we learn more of plant chemistry, we should also examine the effects on herbivores, because plant chemistry alone cannot reveal the fate of the compounds in herbivores.

Eucalypts could be an ideal system in which to test hypotheses that relate oxidative activity to insect herbivory because there is wide variation in oxidative activity between eucalypt species (this study) and between individuals within species (Marsh *et al*., 2017b), a variety of insect herbivores feed on eucalypt foliage (Fox & Morrow, 1981; Paine *et al*., 2011), and herbivory by insects differs between eucalypt species and individuals (Fox & Macauley, 1977; Paine *et al*., 2011; Marsh *et al*., 2017b). If oxidative activity does deter herbivory by some insects, our results suggest that the most likely eucalypts to benefit would be those containing high concentrations of ellagitannins, as well as prodelphinidin subunits in large PAs. Caffeic acid derivatives, such as caffeoyl quinic acids, are also efficiently oxidised at high pH, and by plant oxidative enzymes (Kim *et al*., 2018). These compounds could be important in eucalypts as well, because quinic acid derivatives partially determined the oxidative activity of eucalypt extracts.

The fact that ellagitannin concentrations had strong effects on both protein precipitation capacity and oxidative activity probably explains why there was a positive correlation between the two biological activities. On the surface, this suggests that plants containing high concentrations of ellagitannins might be somewhat protected against both mammalian (through protein binding) and insect (through oxidation) herbivores. However, before making these sorts of assumptions, we need a better understanding of the relationship between specific *in vitro* activity and the *in vivo* effects of tannins on herbivores. This is particularly pertinent given that ellagitannin concentrations did not affect the capacity to reduce *in vitro* N digestibility, even though they affected protein precipitation capacity.

This is the first study to investigate the particular structural features of tannins that correlate with changes in *in vitro* N digestibility, which can affect habitat quality and the reproductive success of marsupial folivores (DeGabriel *et al*., 2009; Youngentob *et al*., 2011). The results suggest that PAs might be particularly important in influencing *in vitro* N digestibility. Despite our expectations, different tannin subgroups affected the capacity to reduce *in vitro* N digestibility relative to *in vitro* protein precipitation capacity. The capacity to reduce *in vitro* N digestibility was strongly positively correlated with the proportion of prodelphinidin subunits in PAs, and the mDP of PAs. Both of these factors have previously been shown to influence protein precipitation capacity generally (e.g. Jones *et al*., 1976; Porter & Woodruffe, 1984; Kumar & Horigome, 1986; Osborne & McNeill, 2001; Lokvam & Kursar, 2005; Huang *et al*., 2011; Saminathan *et al*., 2014), which made it surprising that there was a negative relationship between the protein precipitation capacity of eucalypt samples and the mDP of PAs. Nevertheless, this could be due to a negative correlation between the mDP of PAs and the concentration of late‐eluting HHDP derivatives (data not shown). It would be useful to know what happens to the hydrolysable tannins in samples during the digestible N assay, such as whether they dissociate from protein or hydrolyse in response to the assay conditions, because they clearly precipitate protein in the protein precipitation capacity assay.

The results of our study demonstrate that the biological activity of tannin mixtures in plants is a complex trait relying on several classes of compounds and, probably, many individual structures, as well as the specific conditions and proteins encountered after ingestion by a herbivore. Despite recent breakthroughs in identifying genes underlying some aspects of tannin structure (e.g. Liu *et al*., 2016), there are unlikely to be genes of large effect that explain this biological activity (Kulheim *et al*., 2011). Skovmand *et al*. (2018) argue that the genes responsible for tannin synthesis could act as keystone genes influencing many ecosystem processes, but the complexity of tannin composition in foundation trees, such as eucalypts, suggests that genes of large effect are not likely.

## Conclusions

Our study shows that the tannin composition of plant extracts affects their biological activity. In particular, it is possible to elucidate the broad structural features that contribute to biological activity, even when each individual compound in a complex mixture has not been identified. This is important, because it confirms that the new analytical techniques utilised here could be a valuable tool allowing researchers to understand how the composition of a widespread group of plant secondary metabolites such as the tannins influences the ecology of both individuals and populations of herbivores. It also suggests that future studies that characterise the individual tannins in specific subgroups could provide more detailed insight into the major patterns revealed in the current work.

## Author contributions

WJF, IRW and J‐PS conceived the idea. DN and IRW collected the samples, and KJM and J‐PS conducted the chemical assays. KJM, CK and RC analysed the data. KJM led the writing of the manuscript, with contributions from all other authors.
